# Screening for small for gestational age using third-trimester ultrasound markers: protocol for a systematic review and meta-analysis of screening test accuracy

**DOI:** 10.1186/s13643-018-0885-4

**Published:** 2018-12-03

**Authors:** Cédric Gasse, Kim Paquette, Suzanne Demers, Stéphanie Roberge, Emmanuel Bujold, Amélie Boutin

**Affiliations:** 1 Reproduction, Mother and Child Health Unit, CHU de Québec - Université Laval Research Center, Universite Laval, 2705, boul. Laurier, Quebec, QC G1V 4G2 Canada; 2 Department of Social and Preventive Medicine, Faculty of Medicine, Universite Laval, 1050, avenue de la Medecine, Quebec, QC G1V 0A6 Canada; 3 Department of Gynecology, Obstetrics and Reproduction, Faculty of Medicine, Universite Laval, 1050, avenue de la Médecine, Quebec, QC G1V 0A6 Canada; 4 Harris Birthright Research Centre of Fetal Medicine, Fetal Medicine Research Institute, King’s College Hospital, London, UK; 5 Department of Obstetrics and Gynaecology, Faculty of Medicine, University of British Columbia, 4500 Oak Street, Vancouver, BC V6H 3N1 Canada

**Keywords:** Ultrasound, Small for gestational age, Screening, Screening accuracy, Third trimester

## Abstract

**Background:**

Fetal growth restriction (FGR) is a complication of pregnancy associated with major neonatal morbidity and commonly diagnosed at birth based on birth weight below the 5th or the 10th centile. There is no consensus on the use of routine third-trimester ultrasound for the detection of FGR in a general population. This systematic review aims to estimate the performance of third-trimester ultrasound markers in the screening for babies who are small for gestational age in low-risk or general population.

**Methods:**

A systematic review of screening test accuracy will be conducted. The databases MEDLINE, Embase, Cochrane Library, and Web of Science will be searched from their inception until December 2017, as well as reference lists of included studies and previous related review articles. Studies screening for FGR in a low-risk or general population using third-trimester ultrasound markers and reporting low birth weight for gestational age (small for gestational age at birth) as a reference will be eligible. Two reviewers will independently screen references for inclusion, assess the risk of bias, and extract data. The *Quality Assessment of Diagnostic Accuracy Study 2* (QUADAS-2) tool will be used to assess the methodological quality and validity of individual studies. The hierarchal summary receiver operating characteristic and random effects hierarchal bivariate models (*Bivariate*) will be used to estimate the pooled sensitivity and specificity of each ultrasound marker and to compare the discriminative ability of the different ultrasound markers. Subgroup and sensitivity analyses will be performed to explore the heterogeneity between studies and to assess the effect of screening tests’ characteristics (e.g., timing) on their discriminative ability.

**Discussion:**

This systematic review will determine the relevance of routine third-trimester ultrasound markers in the screening for FGR in low-risk or general population and their usefulness in standard pregnancy care. Additionally, this knowledge synthesis represents a step in the optimization of the discriminative ability of third-trimester ultrasound and predictive tools, allowing for targeted interventions aiming at the reduction of FGR complications and ultimately improving infants’ health.

**Systematic review registration:**

This protocol has been registered at PROSPERO: international prospective register of systematic reviews. The register number is CRD42018085564.

**Electronic supplementary material:**

The online version of this article (10.1186/s13643-018-0885-4) contains supplementary material, which is available to authorized users.

## Background

Fetal growth restriction (FGR) is a complication that affects approximately 5 to 10% of all pregnancies [1]. It is typically defined as an estimated fetal weight or abdominal circumference below the 5th or the 10th centile according to gestational age and sex, with severe cases being below the 3^rd^ centile. It can arise from several maternal, fetal, and/or placental origins, including placental insufficiency or placental abnormality, a genetic abnormality, or an infection during pregnancy [2–5]. FGR is commonly diagnosed at birth, the affected infants having a low birth weight for their gestational age and sex and being small for gestational age (SGA). FGR is associated with premature births, neurodevelopmental delays in children, and about half of all fetal deaths [6–8]. It is also associated with chronic health problems in adulthood, such as diabetes, obesity, and hypertension [9].

Fetal ultrasound is now part of the standard of care during pregnancy, allowing for the prediction, monitoring, and prevention of many complications, such as fetal and placental anomalies, fetal anemia, fetal and placental growth, and cervical length [10–16]. Over the last decade, many ultrasound tools (middle cerebral artery [MCA] Doppler, uterine arteries [UtA] Doppler, umbilical artery [UA] Doppler, cerebroplacental ratio [CPR], ductus venosus [DV] Doppler, etc.) and markers (e.g., vascularization indices) have been developed to detect growth restrictions and to predict short- and long-term complications related to FGR [17–20]. However, there is a clinical equipoise regarding the use of third-trimester fetal ultrasound as a screening tool for FGR.

Despite the potentially significant impact of the new ultrasound technologies, the lack of consensus on the clinical value of these new tools and markers and when to use them in clinical practice has led to significant heterogeneity in clinical guidelines regarding third-trimester ultrasound recommendations [21–27]. Currently, the Canadian [21] and American [22] obstetrics guidelines recommend the use of the third-trimester ultrasound to assess fetal growth only in women with risk factors of FGR. On the other hand, French obstetrics societies recommend a third-trimester ultrasound systematically to all women for monitoring fetal growth and as part of the prevention of fetal deaths [24–26]. The American [22] and European [23] guidelines state that screening for fetal growth abnormalities using physical examination alone can identify only one third of FGR [27]. A recent meta-analysis showed that an ultrasound estimation of birth weight is better than maternal or clinical (including abdominal palpation, fundal height, and Leopold maneuver) estimations [28]. Many experts in the field support the need for the third-trimester ultrasound to assess fetal and maternal well-being [29]. A population-based study showed that antenatally detected FGR was associated with a fivefold lower risk of perinatal mortality than non-detection of FGR [30]. Therefore, it is essential to determine the performance of the third-trimester ultrasound tools and markers and their optimal parameters in order to maximize the benefits for the fetuses and the infants. Thus, this study aims to estimate the performance of third-trimester ultrasound markers in the screening for FGR in a low-risk or general population of pregnant women and to determine the parameters (e.g., timing, thresholds) optimizing the performance of third-trimester ultrasound markers.

## Methods

A systematic review will be conducted to synthesize knowledge on the ability of third-trimester ultrasound markers to identify fetuses at risk of low birth weight for gestational age. The guidelines of the Preferred Reporting Items for Systematic Review and Meta-Analysis Protocols (PRISMA-P) were followed to report the methodology (Additional file 1) [31]. The methodology was developed according to the recommendations of the Cochrane Collaboration for diagnostic test accuracy meta-analyses [32]. The protocol of this systematic review is registered in PROSPERO [CRD42018085564].

### Eligibility criteria

#### Population

Studies of low-risk or general [unselected] population of women with a singleton pregnancy will be eligible. There will be no exclusion based on maternal age. Studies of multi-fetal pregnancies, pregnancies complicated by fetal abnormalities, and studies restricted to specific high-risk populations will be excluded.

#### Index test

All ultrasound markers (fetal biometrics, MCA Doppler, UtA Doppler, UA Doppler, CPR, DV Doppler, renal Doppler, amniotic fluid volume, placental thickness, etc.) measured at any time over the third trimester (28 weeks’ gestation or more) will be eligible. Articles which do not report data on ultrasound markers separately will be excluded (e.g., predictive model combining ultrasound and serum biomarkers without information on ultrasound markers only will be excluded).

#### Reference test

The reference will be the diagnosis of SGA at birth, based on any criteria reported in a study.

#### Outcome

The outcome of interest is the discriminative performance of third-trimester ultrasound markers in the screening for SGA. Articles which do not report any data on discriminative performance [such as a c-statistic, sensitivity and specificity, or true positive (TP), false positive (FP), true negative (TN), and false negative (FN)] will be excluded.

#### Study design

Published clinical trials and cohort studies (prospective and retrospective) which estimated the predictive performance of any third-trimester ultrasound markers will be eligible. There will be no language or publication date restriction. If data from the same sample have been reported in different publications, only the report with the largest study group will be used in the quantitative analyses.

### Information sources

MEDLINE (via PubMed), Embase, Cochrane Library, and Web of Science will be searched from their inception until December 2018 for the identification of eligible studies. Clinical registries (Health Canada’s Trial Registry, ClinicalTrials.gov, Controlled-Trials.org, WHO International Clinical Trials Registry, Cochrane’s CENTRAL) and the grey literature (OpenGrey, TRIP and Health Canada website) will be searched for potentially eligible reports. References of selected articles and previous related review articles will also be screened to identify additional potentially eligible references. Co-authors who are experts in the field will be consulted to review the list of included articles and identify any studies unretrieved by our search of the literature.

### Search strategies

Our search strategies are presented in Table 1 (MEDLINE), Table 2 (Embase), Table 3 (Cochrane Library), and Table 4 (Web of Science). MESH, Emtrees, and free vocabularies about the concepts of “pregnant women,” “ultrasound,” “small for gestational age,” and “predictive value” were used. Terms for specific ultrasound markers and “third trimester” were not included in order to attain a higher sensitivity. For instance, many authors may not have indicated such information in the title or in the abstract or could have used a different term (e.g., they could have indicated the gestational age instead of using the “third trimester” expression). All the authors revised and approved the search strategies. A filter will be used in Embase to reduce the number of duplicates between the Embase and MEDLINE databases ([embase]/lim NOT [medline]/lim).

**Table 1:** Search strategy for MEDLINE (PubMed)

Step	Concept	Search strategy
#1	Population = pregnant women	“Pregnancy”[Mesh: NoExp] OR “Pregnancy Outcome”[Mesh: NoExp] OR “Pregnancy, High-Risk”[Mesh] OR “Pregnancy Trimesters”[Mesh] OR pregnant[TIAB] OR pregnanc*[TIAB] OR gestation*[TIAB] OR fetus[Mesh: NoExp] OR fetal[TIAB] OR foetal[TIAB] OR fetus[TIAB] OR foetus[TIAB] OR foetuses[TIAB] OR fetuses[TIAB]
#2	Index test = ultrasound	“Ultrasonography”[Mesh: NoExp] OR “Ultrasonography, Prenatal”[Mesh: NoExp] OR (“Ultrasonography, Doppler”[Mesh] Not “Echocardiography, Doppler”[Mesh]) OR ultrasonograph*[TIAB] OR ultrasound[TIAB] OR “ultra sound”[TIAB] OR sonograph*[TIAB] OR scan[TIAB] OR Doppler[TIAB] OR echograph*[TIAB]
#3	Reference test = small for gestational age measured by birth weight	“Infant, low birth weight”[Mesh] OR “fetal weight”[Mesh] OR “Birth weight”[Mesh: NoExp] OR “Fetal growth retardation”[Mesh] OR “small for gestational age”[TIAB] OR “SGA”[TIAB] OR “birth weight”[TIAB] OR “birth-weight”[TIAB] OR “birthweight”[TIAB] OR “IUGR”[TIAB] OR “growth restriction”[TIAB] OR “growth retardation”[TIAB] OR “fetal weight”[TIAB] OR “foetal weight”[TIAB] OR “fetus weight”[TIAB] OR “foetus weight”[TIAB] OR “neonatal weight”[TIAB] OR “newborn weight”[TIAB] OR “infant weight”[TIAB] OR “fetal size”[TIAB] OR “foetal size”[TIAB] OR “fetus size”[TIAB] OR “neonatal size”[TIAB] OR “newborn size”[TIAB] OR “infant size”[TIAB] OR “fetal growth”[TIAB] OR “foetal growth”[TIAB] OR “fetus growth”[TIAB] OR “foetus growth”[TIAB] OR “neonatal growth”[TIAB] OR “newborn growth”[TIAB] OR “infant growth”[TIAB]
#4	Outcome = predictive value	“Sensitivity and Specificity”[MeSH] OR “Predictive Value of Tests”[MeSH] OR Sensitivit*[TIAB] OR specificity[TIAB] OR “predictive value*”[TIAB] OR “predictive model*”[TIAB] OR “false-positive rate”[TIAB] OR “detection rate”[TIAB] OR “ROC curve”[TIAB] OR “receiver operating characteristic”[TIAB] OR “AUC”[TIAB] OR “area under the curve”[TIAB] OR “Prenatal Diagnosis”[Mesh: NoExp] OR “Neonatal Screening”[Mesh] OR “Diagnostic imaging”[Subheading] OR screening[TIAB]
#5	Human filter	Animals[MeSH] NOT Humans[MeSH]
#6	Combination of concepts	#1 AND #2 AND #3 AND #4 NOT #5

**Table 2:** Search strategy for Embase

Step	Concept	Search strategy
#1	Population = pregnant women	“Pregnancy”/de OR pregnant:TI,AB,kw OR pregnanc*:TI,AB,kw OR gestation*:TI,AB,kw OR fetus/exp. OR fetal:TI,AB,kw OR foetal:TI,AB,kw OR fetus:TI,AB,kw OR foetus:TI,AB,kw OR foetuses:TI,AB,kw OR fetuses:TI,AB,kw
#2	Index test = ultrasound	“ultrasound”/exp. OR “echography”/de OR “fetus echography”/de OR (“Doppler ultrasonography”/exp. Not “Doppler echocardiography”/exp) OR ultrasonograph*:TI,AB,kw OR ultrasound:TI,AB,kw OR “ultra sound”:TI,AB,kw OR scan:TI,AB,kw OR Doppler:TI,AB,kw OR sonograph*:TI,AB,kw OR echograph*:TI,AB,kw
#3	Reference test = small for gestational age measured by birth weight	“Birth weight”/exp. OR “fetus weight”/exp. OR “intrauterine growth retardation”/de OR “small for date infant”/exp. OR “small for gestational age”:TI,AB,kw OR “small for age infant”:TI,AB,kw OR SGA:TI,AB,kw OR “birth weight”:TI,AB,kw OR birth-weight:TI,AB,kw OR birthweight:TI,AB,kw OR “growth restriction”:TI,AB,kw OR “growth retardation”:TI,AB,kw OR IUGR:TI,AB,kw OR ((fetal OR foetal OR fetus OR foetus OR newborn OR infant OR neonatal) NEAR/2 (weight OR size OR growth)):TI,AB,kw
#4	Outcome = predictive value	“Sensitivity and Specificity”/exp. OR “Predictive Value”/exp. OR Sensitivit*:TI,AB,kw OR specificity:TI,AB,kw OR “predictive value*”:TI,AB,kw OR “predictive model*”:TI,AB,kw OR “false-positive rate”:TI,AB,kw OR “detection rate”:TI,AB,kw OR “ROC curve”:TI,AB,kw OR “receiver operating characteristic”:TI,AB,kw OR “AUC”:TI,AB,kw OR “area under the curve”:TI,AB,kw OR “screening”/de OR “screening test”/exp. OR “Prenatal screening”/exp. OR “Newborn screening”/exp. OR screening:TI,AB,kw
#5	Human filter	“Animal”/exp. NOT “Human”/exp
#6	Filter Embase only	[embase]/lim NOT [medline]/lim
#7	Combination of concepts	#1 AND #2 AND #3 AND #4 AND #6 NOT #5

**Table 3:** Search strategy for Cochrane Library

Step	Concept	Search strategy
#1	Population = pregnant women	“Pregnancy”(MeSH unexploded) OR “Pregnancy Outcome”(MeSH unexploded) OR “Pregnancy, High-Risk”(Mesh) OR “Pregnancy Trimesters”(Mesh) or pregnanc*:ti,ab,kw or gestation*:ti,ab,kw or (MeSH descriptor fetus explode all trees) or fetal:ti,ab,kw or foetal:ti,ab,kw or fetus:ti,ab,kw or foetus:ti,ab,kw or foetuses:ti,ab,kw or fetuses:ti,ab,kw
#2	Index test = ultrasound	“Ultrasonography”(MeSH unexploded) OR “Ultrasonography, Prenatal”(MeSH unexploded) OR(“Ultrasonography, Doppler”(MeSH) Not “Echocardiography, Doppler”(MeSH)) OR ultrasonograph*:ti,ab,kw OR ultrasound:ti,ab,kw OR “ultra sound”: ti,ab,kw OR scan:ti,ab,kw OR Doppler:ti,ab,kw OR sonograph*:ti,ab,kw OR echograph*:ti,ab,kw
#3	Reference test = small for gestational age measured by birth weight	“Infant, low birth weight”(MeSH) OR “fetal weight”(MeSH) OR “Birth weight”(MeSH unexploded) OR “Fetal growth retardation”(MeSH) OR “small for gestational age”:ti,ab,kw OR “small for age infant”:ti,ab,kw OR SGA:ti,ab,kw OR “birth weight”:ti,ab,kw OR birth-weight:ti,ab,kw OR birthweight:ti,ab,kw OR “growth restriction”:ti,ab,kw OR “growth retardation”:ti,ab,kw OR IUGR:ti,ab,kw OR “fetal weight”:ti,ab,kw OR “foetal weight”:ti,ab,kw OR “fetus weight”:ti,ab,kw OR “foetus weight”:ti,ab,kw OR “neonatal weight”:ti,ab,kw OR “newborn weight”:ti,ab,kw OR “infant weight”:ti,ab,kw OR “fetal size”:ti,ab,kw OR “foetal size”:ti,ab,kw OR “fetus size”:ti,ab,kw OR “neonatal size”:ti,ab,kw OR “newborn size”:ti,ab,kw OR “infant size”:ti,ab,kw OR “fetal growth”:ti,ab,kw OR “foetal growth”:ti,ab,kw OR “fetus growth”:ti,ab,kw OR “foetus growth”:ti,ab,kw OR “neonatal growth”:ti,ab,kw OR “newborn growth”:ti,ab,kw OR “infant growth”:ti,ab,kw
#4	Outcome = predictive value	“Sensitivity and Specificity”(MeSH) OR “Predictive Value of Tests”(MeSH) OR Sensitivit*:ti,ab,kw OR specificity:ti,ab,kw OR “predictive value*”:ti,ab,kw OR “predictive model*”:ti,ab,kw OR “false-positive rate”:ti,ab,kw OR “detection rate”:ti,ab,kw OR “ROC curve”:ti,ab,kw OR “receiver operating characteristic”:ti,ab,kw OR “AUC”:ti,ab,kw OR “area under the curve”:ti,ab,kw OR “screening”(MeSH unexploded) OR “screening test”(MeSH) OR “Prenatal screening”(MeSH) OR “Newborn screening”(MeSH) OR screening:ti,ab,kw
#5	Human filter	“Animal”(MeSH) NOT “Human”(MeSH)
#6	Combination of concepts	#1 AND #2 AND #3 AND #4 NOT #5

**Table 4:** Search strategy for Web of Science

Step	Concept	Search strategy
#1	Population = pregnant women	TS=(pregnant OR pregnanc* OR gestation* OR fetal OR foetal OR fetus OR foetus OR foetuses OR fetuses) OR TI=(pregnant OR pregnanc* OR gestation* OR fetal OR foetal OR fetus OR foetus OR foetuses OR fetuses)
#2	Index test = ultrasound	TS=(ultrasonograph* OR ultrasound OR “ultra sound” OR scan OR Doppler OR sonograph* OR echograph*) OR TI=(ultrasonograph* OR ultrasound OR “ultra sound” OR scan OR Doppler OR sonograph* OR echograph*)
#3	Reference test = small for gestational age measured by birth weight	TS=(“small for gestational age” OR “small for age infant” OR SGA OR “birth weight” OR birth-weight OR birthweight OR “growth restriction” OR “growth retardation” OR IUGR OR ((fetal OR foetal OR fetus OR foetus OR newborn OR infant OR neonatal) NEAR/2 (weight OR size OR growth))) OR TI=(“small for gestational age” OR “small for age infant” OR SGA OR “birth weight” OR birth-weight OR birthweight OR “growth restriction” OR “growth retardation” OR IUGR OR ((fetal OR foetal OR fetus OR foetus OR newborn OR infant OR neonatal) NEAR/2 (weight OR size OR growth)))
#4	Outcome = predictive value	TS=(Sensitivit* OR specificity OR “predictive value*” OR “predictive model*” OR “false-positive rate” OR “detection rate” OR “ROC curve” OR “receiver operating characteristic” OR “AUC” OR “area under the curve” OR screening)
#5	Human filter	TS=(Microorganisms OR Plants OR Invertebrates OR Protochordates OR “Nonhuman Vertebrates”)
#6	Combination of concepts	#1 AND #2 AND #3 AND #4 NOT #5

### Selection process

The EndNote X8 software (Thomson Reuters® EndNote X8 for Mac 1988–2017, version 18.2.0.13302) will be used to manage all the references retrieved from the electronic databases and to remove duplicates. The remaining references will be exported to Excel (Microsoft® Excel® for Mac 2011, version 14.7.3) to conduct the selection process. Two independent reviewers (CG, KP) will screen the titles and abstracts to assess the eligibility of studies, followed by a full-text evaluation of the remaining articles’ eligibility. If no consensus is reached between the two reviewers regarding the eligibility of a study, a third independent reviewer with expertise in perinatal epidemiology will be consulted (EB).

### Data extraction

Two reviewers (CG, KP) will independently extract data from the selected articles using a standardized data collection form and an Excel sheet. The data collection form will be piloted using three randomly selected articles and will be corrected to improve completeness, clarity, and usability, as needed. Any discordance between the two data collections will be resolved by consensus. A third independent reviewer (EB) will be consulted in case of persistent disagreement. Authors will be contacted if some data are missing and needed to conduct the analyses.

Extracted data will include (1) study characteristics (authors, study design, year of conduction and publication, country, clinical setting, sample size, participation rate, lost to follow-up rate, number of exclusion), (2) characteristics of participants (maternal age, parity, ethnicity, fetal sex), (3) characteristics of the screening tools (ultrasound markers used, threshold for each ultrasound markers, gestational age at ultrasound exam, type of the device used, time between ultrasound exam and delivery, personnel conducting the exam), (4) characteristics of the reference (definition of SGA, number of SGA, reference curve used for the classification of SGA), (5) performance data ([TP, FP, TN, and FN; sensitivity, specificity, area under the curve (AUC)]), and (6) other variables (birth weight, gestational age at birth).

### Risk of bias in individual studies

Using the Quality Assessment of Diagnostic Accuracy Study 2 (QUADAS-2) tool [33], the risk of bias and concerns about applicability will be assessed for each included study by two reviews independently. The QUADAS-2 assesses four different components of bias, which are related to the patient selection, the index test, the reference test, and the flow and timing. The presence of one domain at high risk of bias will translate in an overall high risk of bias.

### Statistical analysis and data synthesis

The TP, FP, TN, and FN will be used to compute the sensitivity and specificity of each marker in all studies. The corresponding sensitivity and specificity will be presented in a coupled forest plot.

As recommended by the Cochrane Collaboration [32], *hierarchal summary receiver operating characteristic* (HSROC) [34, 35] and random effects hierarchal bivariate models (*Bivariate*) [36] will be used to pool the results. As variation in thresholds and accuracy of the markers across studies are expected, for each SGA definition where there will be at least three studies with the same definition, HSROC model will be performed to obtain summary ROC curves for each marker. If a study presents more than one threshold, the most commonly used one among the studies included in our meta-analyses will be selected. For each marker with at least three studies with the same threshold, the *Bivariate* model will estimate the mean sensitivity and specificity for the specific thresholds with their 95% confidence region and 95% prediction region.

Indirect comparison of each marker will be done using a *HSROC* meta-regression model including the different markers as a covariate to compare their screening test accuracy for SGA.

Cochrane Review Manager (version 5.1, The Cochrane Collaboration, 2011) and the statistical software packages SAS (Version 9.3, SAS Institute Inc., Cary, NC, USA) will be used to complete the analyses.

### Additional analysis

Subgroup and sensitivity analyses will be conducted to explore the possible heterogeneity between studies. Subgroup analyses will be performed to observe the effect of the screening test characteristics (gestational age at screening, timing of screening before delivery, type of device used [2D vs 3D ultrasound], study date, ethnicity,) on the discriminative ability of third-trimester ultrasound markers. Also, sensitivity analyses by study design and restricted to studies at low risk of bias will be done.

### Strength of evidence

The quality and evidence of this review will be assessed using the *Grading of Recommendations, Assessment, Development and Evaluation* (GRADE) domains (study limitations, consistency of effect, imprecision, indirectness, publication bias, large magnitude of effect, dose-response gradient, residual confounding effect).

## Discussion

There is no consensus on the recommendation of a third-trimester ultrasound for the assessment of fetal growth in a general population. This systematic review will synthesize the published evidence on the performance of third-trimester ultrasound markers in the screening for FGR in low-risk or general populations. The gathered knowledge will participate in the evaluation of the usefulness of a routine third-trimester ultrasound examination as part of standard antenatal visits schedule, and if any marker is deemed useful, the review will allow for the optimization of the third-trimester ultrasound screening process. Thus, this study will help to determine the tools allowing for the identification of pregnancies at higher risk of SGA which could benefit from closer monitoring and targeted interventions. Considering the frequency and potentially serious complications of FGR, this knowledge synthesis can significantly contribute to the improvement of the health of fetuses and infants.

## Additional file

Additional file 1: PRISMA-P. (PDF 100 kb)

## Abbreviation

AUC: Area under the curve
CPR: Cerebroplacental ratio
DV: Ductus venosus
FGR: Fetal growth restriction
FN: False negative
FP: False positive
HSROC: Hierarchal summary receiver operating characteristic
MCA: Middle cerebral artery
QUADAS-2: Quality Assessment of Diagnostic Accuracy Study 2
SGA: Small for gestational age
TN: True negative
TP: True positive
UA: Umbilical artery
UtA: Uterine arteries
